# Supplementary material for: Are carbon emissions trading and green financial instruments synergistic? -Comprehensive quantitative research based on content analysis
Source: PLoS One. 2024 Mar 7;19(3):e0298601. doi: 10.1371/journal.pone.0298601 (PMC10919601; doi:10.1371/journal.pone.0298601)
Supplement: S1 Data — (ZIP) [file pone.0298601.s001.zip › Data/地级市Z_F工作报告.zip!/╣π╬≈/╙±┴╓╩╨/└┤╫╘╣1⁄2╓┌║┼í╛┬φ┐╦╩2╛▌═°í┐_2013.docx]

一、2012 年工作回顾

2012 年，在自治区党委、政府和市委的领导下，我们认真贯彻落实党的十八大精神，以科学发展观为指导，抢抓机遇，克难攻坚，深化统筹城乡综合配套改革，加快创建“六个示范”，克服了经济下行压力加大、要素制约突出等困难，保持了经济社会平稳较快发展的良好势头，圆满完成了全年各项目标任务。

——经济增长速度加快。全市实现地区生产总值1120.48 亿元，增长11%；全社会固定资产投资突破1000亿元，达1004.26亿元，增长26.8%；规模以上工业总产值突破1000 亿元，达1056.4 亿元，增长14.5%；财政收入突破100亿元，达100.36亿元，增长16.9%；金融机构本外币存款余额突破1000亿元，达1042.79 亿元，增长15.78%；有9 项主要经济指标增幅高于全区水平。

——先行先试成果显著。统筹城乡综合配套改革试验工作不断深化，城乡发展更加协调。城镇居民人均可支配收入22171元，增长13.17%，比全区水平高928 元，增幅排全区第3 位；农民人均纯收入7269元，增长15.9%，比全区水平高1261元。城乡居民收入比由上年的3.12∶1缩小到3.05∶1。城镇化率达43.6%，比上年提高2.1个百分点。

——产业升级有力推进。完成技改投资435.24 亿元，增长42.7%；实现规模以上工业增加值323.35亿元，增长18.2%。新增亿元工业企业55家、规模以上工业企业28家。食品健康产业总产值达到200亿元。

——重大项目实现突破。新开工重大项目290 个，竣工160个，完成投资480.34亿元。玉铁高速公路即将通车，玉铁铁路实现铺轨，新华联50 万吨精制镍铁及深加工项目获得批准。

——发展优势不断凸显。玉柴发动机市场占有率继续保持全国行业第一。畜禽主导产业养殖总量保持全区第一；农业产业化水平和超级稻推广居全区第一；家电下乡销售总额居全区第一。城镇污水集中处理率、生活垃圾无害化处理率居全区前列；城乡建设用地增减挂钩工作居全区前列。被确定为全国发展改革试点城市、全国首批“创新社会管理示范基地”、国家低丘缓坡荒滩土地综合开发利用试点城市。北流市成为国家外贸转型升级专业示范基地，被评为全国农田水利基本建设先进单位；容县被评为全国国土资源节约集约模范县和全国计划生育优质服务先进县；博白县成为中国民间文化艺术之乡，被评为全国农村集体“三资”管理示范县；陆川县被评为全国粮食生产先进县；兴业县成为国家现代农业示范区。

这些成绩的取得，标志着我们在富民强市新跨越的道路上迈出了新的更加坚实的步伐！

一年来，我们主要做了以下工作：

（一）突出统筹城乡发展。7个自治区级土地综合整治项目顺利推进，完成土地整理7.4万亩。新增农村承包经营土地流转15.28万亩，流转率比上年提高5.75个百分点。全面实施居住证制度，接纳进城务工人员随迁子女2.47万人入学，增长34.8%。撤乡建镇3个，撤村建居11个，新建社区27个。110个乡镇（街道）建立了农村集体“三资”委托服务中心；林权流转面积22.74万亩。新（改）建农村公路1435公里，完成投资4.39亿元，新增133个行政村通硬化路，硬化率达90.3%。建成人饮工程项目150个，完成投资3.6亿元，解决45.41万人饮水安全问题。投资3.51亿元，建设农村幼儿园208个，维修改造农村义务教育学校546所。建成116个村级公共服务中心和299个“农家书屋”。下放扩权强县审批事项460项，县域经济加快发展，玉州、北流、博白财政收入均超过10亿元。

（二）突出产业转型升级。机械产业稳步发展，水泥陶瓷、食品健康、有色金属、服装皮革、电子信息等产业发展提速。大容山风电场、玉柴厂区连片光伏发电、凯迪生物质能发电等项目顺利推进。组织实施11项广西千亿元产业重大科技攻关工程重大专项，玉柴获得国家科技进步二等奖、首届自治区主席质量奖，玉柴重工、三环集团成为自治区级创新型企业，广西银亿、参皇集团成为自治区级创新型试点企业。实施农业产业“123工程”，实现农林牧渔业总产值384.35亿元，增长6.34%。粮食总产188.96万吨，增长5.92%，增幅高于全国、全区水平。新增4个国家级、13个自治区级畜禽养殖标准化示范场。林业、中药材等特色产业全面增长。毅德国际商贸城、宏进市场二期等重点商贸物流项目加快推进。交通银行玉林分行开业。完成旅游项目51个，国内旅游收入88.21亿元，增长31.76%。实现社会消费品零售总额422.83亿元，增长16.54%，增幅排全区第4位。

（三）突出重大项目建设。统筹推进投资3000万元以上的重大项目860个。争取中央及自治区项目1131个，资金15.12亿元。引进民间投资792.13亿元，增长29.85%。玉林至陆川二级公路大修竣工，玉林至博白二级公路改造加快推进。开工建设500千伏变电站1座，建成220千伏变电站1座、110千伏变电站2座。开工建设137座水库除险加固工程，完成中小河流治理工程项目10个。玉林国家内燃机及零部件产品质量监督检验中心、新高盛陶瓷、银亿镁盐二期、永耀玻璃、澳通矿业二期等项目竣工投产，德国米勒电动汽车整车生产项目、新翰机械、兴业三一重工、旺仔牛奶、福绵国际服装贸易城等项目顺利推进。

（四）突出城镇化建设。新开工城建项目108个，续建项目84个，完成市政公用设施建设投资64.3亿元，增长39%。一环路提质改造等项目顺利完成，迎宾大道三期、二环南路、玉福大道等10条城市干道加快推进，市文化艺术中心、湿地公园、污水干线管道设施工程等项目进展顺利，玉林城区建成区面积扩大到64平方公里，人口增加到65万人。各县（市）城区建设步伐加快，博白县绿珠大道西段、容县绣江北岸绿带景观工程、陆川县松鹤公园等项目竣工，北流市会仙河公园、兴业县环城大道等项目加快推进。镇、村建设完成投资36.43亿元，增长9%。

（五）突出改革开放合作。实现国家基本药物制度和综合改革全覆盖。完成9个国有文艺院团改革。落实鼓励全民创业的各项政策措施，为中小企业新增担保贷款14.17亿元，新增微型企业3603家，总数达6719家，占全区的17.7%。成功举办了第九届玉博会、第四届药博会、第二届陶博会和第二届广西名特优农产品交易会。玉博会和药博会分别被评为中国十大品牌展览会、中国优秀品牌展览会，获得“中国优秀会展城市”称号和全国“城市品牌建设奖”。主动融入北部湾经济区、西江经济带建设，加强与长三角、珠三角和台湾地区的交流合作，大力推进东部产业转移示范走廊建设，招商引资新签项目565个，投资总额552.83亿元，增长28.07%。外贸出口总额3.58亿美元，增长5.2%。

（六）突出生态文明建设。万元GDP能耗同比下降5%。大力推进重点行业、水源地、养殖企业污染整治和农村环境集中连片整治。全面完成污染物总量减排4项约束性指标任务。主要江河达到相应功能区水质标准，城区主要饮用水源水质长期达标，空气环境质量保持国家二级标准。深入实施“绿满八桂”工程，持续开展“绿化日”植树活动，造林绿化23.68万亩，城区绿化覆盖率40.46%，比上年提高2.46个百分点。

（七）突出民生工程建设。安排142.2亿元投向民生事业，比上年增长19.9%，占公共财政预算支出的75%。为民办的10件实事全面完成。新增农村劳动力转移就业6.46万人、城镇就业4.51万人，城镇登记失业率3.63%。实现城乡居民养老保险全覆盖。160个五保村全部竣工。建成保障性住房7311套，完成投资8.37亿元；改造农村危房9930户，完成投资4.15亿元。新一轮扶贫开发首批整村推进贫困村130个，完成投资1.1亿元，建成项目1031个，惠及群众62.3万人。物价总水平保持平稳。

（八）突出社会事业发展。各级财政对教育投入持续增长，特殊教育学校全部建成，玉东小学、北流市实验中学建成招生，玉州区八中、玉林高中新校区、万秀小学、新石棠小学等学校开工建设，义务教育巩固率达96.03%。玉林师范学院发展取得新成绩。公共图书馆、文化馆（站）、博物馆实现免费开放；实施文艺创作奖和签约文艺家制度，首批签约文艺家15名；参加自治区“八桂群星奖”音乐、舞蹈、戏剧类比赛获得3金4银6铜的好成绩；2家文化企业被列为自治区文化产业示范基地。获授权专利687件，增长31.86%。医疗卫生服务水平不断提高。食品药品安全监管全面加强。城乡公共体育设施不断完善，全民健身活动深入开展。诚信计生有效推进，人口计生工作健康发展。民族、宗教、外事、侨务、对台事务、人防、统计、供销、地震、无线电管理、农机、方志、档案、机构编制、国资监管、机关事务、新闻出版、发展研究、住房公积金管理、社会科学、妇女儿童、老龄、残疾人、科普等工作取得新成绩。

（九）突出社会管理创新。建立完善“一大五小”社会服务管理平台，深入开展社会矛盾“大排查、大防控、大调处、大化解”活动，认真做好群众来信来访工作，民间纠纷调解成功率达97.6%，群众来信来访下降19.5%。筹措资金6亿元，建成市应急指挥中心，推进“天网工程”和城市数字防控系统二期工程建设，社会治安防控体系不断完善。加大社会治安综合治理力度，严厉打击各种违法犯罪活动，侦破了一批大案要案，刑事案件破案率比上年提高25.8%。安全生产形势持续平稳好转。

（十）突出政府自身建设。加强民主法制建设。坚决贯彻执行市委的决策部署，认真执行市人大及其常委会的决议、决定，坚持重大事项向市人大及其常委会报告、向市政协通报制度，自觉接受人大、政协和社会监督，人大代表议案、建议和政协委员提案全部办结。认真听取各民主党派、工商联、无党派人士、各人民团体和社会各界的意见。加强政务服务体系建设，率先在全区实现乡镇政务服务全覆盖，政务服务中心受理、办结事项均居全区第一。建成公共资源交易中心并规范运行。加快机关绩效管理体系建设，强化政务公开和信息公开，加强行政监察和审计监督，各级政府执行力和公信力进一步提高。

国防教育和国防后备力量建设不断加强，驻玉部队在支持地方建设上作出了重要贡献。中直、区直驻玉各单位为经济社会发展提供了大力支持。工会、共青团、妇联等群团组织重要作用得到充分发挥。

二、全面建成小康社会的要求和2013年目标任务

党的十八大提出到2020年实现全面建成小康社会的宏伟目标。与全区全国同步全面建成小康社会，是历史赋予我们的光荣使命。当前，世界经济进入深度转型调整期，低速增长态势仍将延续，我国发展仍面临不少风险和挑战，不平衡、不协调、不可持续问题依然突出，我市发展面临的困难明显增大。同时，我们也应看到，我国仍处于重要战略机遇期，经济社会发展的基本面长期趋好。我市统筹城乡综合配套改革不断深化，工业化、城镇化快速发展，具备在较长时期内实现经济持续健康发展的基础和条件。我们要抢抓机遇，奋发有为，全面做好今年的各项工作，在全面建成小康社会征途上奋勇前进。

与全区全国同步全面建成小康社会的目标：2013年~2020年，全市地区生产总值年均增长12.1%，预计2020年达到3710亿元，比2010年翻2.14番；城镇居民人均可支配收入年均增长11.8%，预计2020年达到5.42万元，比2010年翻1.62番；农民人均纯收入年均增长14.8%，预计2020年达到2.17万元，比2010年翻2.03番。

2013年政府工作的总体要求：全面贯彻落实党的十八大精神，高举中国特色社会主义伟大旗帜，坚持以邓小平理论、“三个代表”重要思想、科学发展观为指导，紧紧围绕主题主线，以提高经济增长质量和效益为中心，稳中求进，开拓创新，大力推进统筹城乡综合配套改革，加快创建“六个示范”，大力推进经济结构战略性调整，增强经济发展的内生活力和动力，大力推进新型工业化、信息化、城镇化、农业现代化，实现经济持续健康发展，大力推进民生工程，着力保障和改善民生，保持社会和谐稳定，为实现富民强市新跨越、与全区全国同步全面建成小康社会打下坚实基础。

今年经济社会发展的主要预期目标：地区生产总值增长11%；财政收入增长12%；规模以上工业增加值增长18%；全社会固定资产投资增长20%；社会消费品零售总额增长15%；外贸进出口总额增长10%；城镇居民人均可支配收入增长13%；农民人均纯收入增长14%；节能减排控制在自治区下达任务内；居民消费价格指数涨幅控制在4%左右；城镇登记失业率控制在4%以内；人口自然增长率控制在自治区下达指标以内。

全面实现今年的发展目标，我们必须以科学发展为主题，以加快转变经济发展方式为主线；必须坚持稳中求进的总基调；必须以提高经济增长质量和效益为中心；必须坚持以扩大内需为战略基点；必须大力实施创新驱动战略；必须坚持解放思想，深化改革开放；必须切实保障和改善民生。

三、2013年工作安排

为实现今年的目标任务，主要抓好以下重点工作：

（一）坚持城乡统筹，着力推进统筹城乡综合配套改革。

深化土地管理制度改革。以7个自治区级试点项目为带动，大力推进农村土地综合整治。建立健全县、乡、村三级土地流转服务中心（站）和纠纷调解中心（室），加大土地流转奖励扶持力度，促进土地向农业实体经济和种养大户集中，力争新增耕地流转面积22万亩，流转率达到38%，土地规模经营率达到30%。制订出台农村集体建设用地使用权交易办法，加快建立城乡统一的建设用地市场。

深化户籍管理制度改革。全面放开城镇落户条件，放开城乡居民市内自由迁徙限制。允许转户农民在过渡期限内保留土地承包经营权、宅基地使用权等农村土地权益，并保留原户籍地计划生育政策、农民种粮直补和与土地相结合的各种补贴。同步落实转户入城人员的就业、医疗、教育、住房、社会保障待遇，确保进城农民转户后享有与城镇居民同等的权益，有序推进农业转移人口市民化。力争新增城镇居民20万人以上。

深化行政区划体制改革。按照自治区主体功能区规划，推进都市型功能区建设，重点建设玉东新区、玉柴新区和龙潭新区，各县（市、区）规划建设1~2个功能区，打造一批新型城镇化和新型工业化示范区、综合配套改革先行区。积极推进撤镇改街、撤村改居，力争县城所在地的镇全部改制为街道办事处，对条件较好的城郊农村改为社区。大力实施村庄整治和旧村改造，促进自然村向中心村集聚、中心村向新型社区转变。

深化农村产权制度改革。推进农村“六权”确权登记颁证工作。建立农村产权管理交易服务中心、农村集体“三资”委托代理服务中心和“三资”信息电子监察系统，促进农村产权有序、规范流转，实现农村产权增值。推进农村集体经济股改，引导组建村级股份经济合作社、土地流转合作社和农民专业合作社，促进农村集体经济发展壮大和农民持续增收。

加快推进“七个一体化”。坚持以政府服务为主导，以市场手段引导生产要素合理流动，推进城乡规划管理、产业发展、基础设施建设管理、基本公共服务、就业和社会保障、社会管理、政策措施一体化，促进城乡基本公共服务均等化。

（二）坚持工业主导，着力推进产业结构优化升级。

完善工业产业体系。加快玉柴船用柴油机、气体发动机、玉柴重工挖掘机零配件、兴业三一重工等项目建设，推动机械产业转型升级，打造千亿元产业。大力发展建筑陶瓷、工艺陶瓷、新型薄板陶瓷、泡沫陶瓷，提升陶瓷产业整体素质。加快发展食品健康、服装皮革、电子信息等传统产业，推动产业集聚发展。大力推进新华联50万吨精制镍铁及深加工、再生资源园区等项目建设，培育发展有色金属、再生资源加工利用等循环经济产业。加快大容山风电场、玉柴厂区连片光伏发电、凯迪生物质能发电、磷酸铁锂动力电池、“三元材料”等项目建设，发展新能源、新材料等战略性新兴产业。

推动企业做大做强。深入实施“强柴兴玉”战略，加快打造千亿玉柴。大力推进“抓大壮小扶微”工程，加强对优质骨干企业的帮扶和服务，培育发展一批百亿元、十亿元企业。通过技术改造、联合重组、招商引资等方式，把一批中小企业培育发展成为亿元企业或规模以上企业。大力发展微型企业。力争新增亿元工业企业30家以上、规模以上工业企业50家以上、微型企业2600家以上。

增强自主创新能力。规划建设玉林市高新技术产业区，加快清华·启迪（玉林）科技园等创新平台建设，实施重大科技攻关项目，打造一批创新型企业和国家高新技术企业。支持玉林国家内燃机及零部件产品质量监督检验中心（二期）、高效节能环保内燃机国家工程实验室、国家级陶瓷检测重点实验室等国家级、自治区级技术机构建设。引导和扶持企业加快设备更新改造，深化“质量兴市”活动，提高技术工艺水平和产品质量，力争技改投资完成544亿元以上。

推进工业向园区集中。实施园区基础设施建设工程，完善园区配套服务设施。重点建设玉柴工业园、龙潭产业园等特色园区，着力打造千亿元、百亿元园区。大力创建自治区级、国家级生态循环产业转移基地、新型工业化产业示范基地，推进新型装备制造示范基地建设，力争园区工业总产值达1120亿元。

推动“两化”融合发展。加快“两化”融合示范项目建设，大力发展电子信息、工业电子应用、机电智能化等新一代技术产业，深化信息技术在工业领域的集成应用。加快培育发展信息化企业。大力推进“数字玉林”和“数字城市”建设。

（三）坚持农业基础地位，着力推进现代农业发展。

高度重视粮食生产。加快高标准基本农田建设，重点推进国家级、自治区级粮食高产示范基地建设。积极发展薯类和豆类等粮食作物。力争粮食播种面积保持在489万亩以上，超级稻种植面积超过200万亩，粮食总产188万吨以上。

发展壮大优势产业。加快创建现代农业示范市。深入实施农业产业“123工程”，大力发展畜牧水产、林业、中药材、粮食、蔬菜、水果等特色优势产业。壮大马铃薯、花卉、桑蚕、食用菌产业，提高单产效益。实施林下经济“千万林农千元增收”工程，推广林果、林菌、林药、林畜、林禽、林蜂等模式，建设林下经济产业带，力争实现林下经济产值80亿元以上。

着力培育农业品牌。加强农业科技研发和先进适用农业技术推广应用，完善良种培育和种业体系，加强农产品质量安全体系建设，积极发展无公害、绿色、有机农产品。加快海峡两岸（广西玉林）农业合作试验区建设，积极引进农业新品种、新技术，建设特色标准化生产示范基地。大力扶持农业龙头企业、农民专业合作社创建品牌，推进“一镇一业”、“一村一品”建设，打造玉林农产品知名品牌。

推动农业集聚发展。培育发展农业龙头企业、农民专业合作社和产业化经营组织，推进土地向规模经营集中，加快农业产业化示范基地建设，力争建设千亩以上农业产业化示范基地14个。大力发展农产品加工业和观光农业、休闲农业、会展农业，推动农超、农企对接，推进农业向“接二（产）连三（产）”延伸。

夯实农业农村基础。继续实施农村饮水安全工程，解决50万农村人口饮水安全问题。加快基本农田建设、农田灌溉设施节水改造。实施水土保持综合治理和山洪灾害防治项目。加快推进农业机械化，大力推广农机新机具、新技术，力争农业耕种收综合机械化水平达40%。强化动植物疫病、农业气象灾害防控工作。

（四）坚持民生优先，着力推进城乡居民收入倍增计划。

实施农民收入倍增计划。大力扶持农民创业，对农民创办的投资额在20万元以下、从业人员20人以下的企业，享受小微企业扶持政策，促进农民创业增收。积极发展农民专业合作社，支持农业龙头企业拓展“公司+农户”模式，实行订单生产、连锁经营，增加农民经营收入。大力发展劳务经济，加强农村劳动力转移就业培训，力争新增农村劳动力转移就业5.4万人，增加农民工资收入。认真落实种粮、良种、农资、生态等各项直补政策，完善农村“一事一议”筹资筹劳办法，提高农村社会保障水平，新农保月人均基础养老金标准比2012年提高20元，农村低保对象月人均补助水平比2012年提高10元，新农合筹资标准由人均290元提高到340元，增加农民转移性收入。推进农村产权合法流转，鼓励农民通过土地经营权、资金、资产入股等方式，建立农村合作经济组织，增加农民财产性收入。完善被征地农民社会保障政策。深入推进新一轮扶贫开发攻坚战，力争建设4 个“十百千”特色产业扶贫示范基地，创建30个产业扶贫示范村。加大对革命老区、水库移民安置区的扶持力度，突出抓好基础设施建设。

实施城镇居民收入倍增计划。落实鼓励民间投资和民营经济发展的政策措施，促进全民创业，大力发展微型企业，增加居民经营收入。高质量做好就业工作，加大对就业困难群体的就业援助力度，确保“零就业家庭”至少有1人就业，力争新增城镇就业2.5万人。完善企业工资正常增长机制，今年最低工资标准提高15％以上。完善义务教育学校、公共卫生与基层医疗卫生事业单位绩效工资制度，加快推进其他事业单位实施绩效工资，推动居民工资收入稳定增长。提高城镇社会保障水平，城镇居民医保补助标准由2012年的每人240元提高到280元，城镇低保对象月人均补助水平比2012年提高20元。提高城镇企业职工基本养老保险待遇。

（五）坚持城镇化带动，着力推进城镇化跨越发展。

抓好中心城市建设。加快推进“玉—北—福”一体化，按照开发玉东、优化城西、拓展南北、完善江南、提升城中的建设布局，建设一批城市主干道、公共建筑、生态绿化工程，拉开城市框架，完善城市功能，优化城市环境。抓好人民东路延长线、教育东路延长线、玉福大道、二环南路、文体路、清宁路、一环路提升改造二期等道路建设；启动建设二环西路、江滨路西段、玉发大道、胜利路、三环东路等城市道路。推进南流江景观长廊、牛运岭公园、西郊公园、城东公园、供水管网改造等市政设施建设。加快玉东新区、玉柴新区和龙潭新区开发建设步伐，增强新区承载能力，促进产业和人口加快集聚。

抓好县城建设。坚持城镇差异化、特色化发展，按照产城融合、区城同建的要求，统筹推进各县（市）城区和产业园区基础设施建设，强化产业支撑，扩大城区规模，增加城区人口，构建以玉林城区为中心，北流、博白城区为次中心，其他县城区为重要节点的桂东南城市群，促进城市组团发展，力争城镇化率提高2.2个百分点以上。

抓好中心镇建设。加大财政对城镇化的投入，城镇土地收益重点用于城镇基础设施建设，增强中心镇承载能力。积极推动中心镇进入自治区扩权强镇试点，培育一批经济强镇。

提高城市品位。推进文明城市创建工作。加快城市供排水、燃气、照明、地下管线等市政设施规划建设，进一步完善便民设施。优先发展公共交通，加快建设城区智能交通管理服务系统。抓好城市重要节点建设，加快城中村改造。完善市容环境综合整治长效机制，推进城乡清洁工程向乡镇、城中村和城乡结合部延伸。继续实施城乡风貌改造工程，大力创建广西名镇名村。

（六）坚持投资拉动，着力推进重大项目加快建设。

统筹推进重大项目建设。实施重大项目890个，年内计划投资550亿元。其中，新开工重大项目300个，年内计划投资260亿元；续建重大项目490个，年内计划投资290亿元；竣工投产重大项目170个；前期工作重大项目100个。

加快交通基础设施建设。建成玉林至铁山港铁路；开工建设玉林火车站改造项目；完成火车站煤场搬迁工程；争取国家批准实施黎湛铁路玉林至贵港段提速改造，推进洛湛铁路永州至玉林段电气化改造、沙河至山口铁路、张家界—桂林—玉林—海口铁路项目前期工作。争取开工建设荔浦至玉林高速公路，做好南宁（苏圩）至北流（清湾）、松旺至铁山港东岸高速公路前期工作；加快岑溪南渡至陆川、贵港木格至博白水鸣等公路建设；新建通行政村硬化路150公里，力争实现93%以上行政村通硬化路。配合抓好铁山港东岸码头以及进港道路前期工作。

加快能源水利设施建设。推进华润风电场、国电风电场、博白那林风电场、玉林500千伏输变电工程、中石油油库二期等项目建设。积极完善主电网、县城电网和农村电网。推进小（二）型病险水库和大中水闸除险加固工程建设。抓好南流江干流、北流河防洪工程建设。加快28个中小河流治理工程项目建设。

积极破解项目建设瓶颈。坚持节约集约用地，依法处置闲置用地，打击违法用地和违法建设。加强政银企合作，力争新增贷款90亿元。规范政府投融资平台，鼓励企业通过资本市场融资。

（七）坚持扩大内需，着力推进消费与服务业同步提升。

加快发展商贸物流业。大力推进玉林无水港、交通商贸物流园、北流龙翔农贸物流市场等10大商贸物流项目建设，提升玉林中药材市场等10个百十亿元专业市场辐射能力，增强“全国流通领域现代物流示范城市”品牌效应，打造广西现代商贸物流基地。加快商务商业区建设，发展一批限额以上住宿餐饮企业和批发零售企业。继续实施万村千乡市场工程和新网工程，新建改造一批集贸批发市场，完善城乡市场流通体系。

加快发展文化产业。鼓励文化企业在与制造、科技、金融、旅游等产业融合中创新业态，延伸文化产业链。加快发展文化娱乐演艺服务业、文化旅游与会展节庆业、工艺美术品与文化用品生产经营业、印刷包装装潢业、文化传媒与文化创意业、文化艺术品与文博经营业等六大文化产业。推进市文化产业园和文化旅游商贸城规划建设，培育发展文化产业集群，培植地方特色文化产业品牌。

加快发展旅游业。推进大容山生态旅游区、六万山生态旅游区、陆川县岭南客家温泉文化城、鹿峰山景区、北流会仙河湿地公园、福绵梦幻水乡景区等项目建设。大力发展乡村旅游业。加快发展旅游企业，整合旅游资源，提高旅游服务配套水平，努力打造旅游目的地城市。

积极扩大消费需求。落实鼓励消费的各项政策，开展美食节、服装节、家装节、汽车展、商品展等促消费活动。积极发展网络、信息、健身、养老、家政等新型消费。加强居民消费市场监督管理，切实维护消费者权益。

（八）坚持扩大开放，着力推进改革创新和区域合作。

大力扶持民营经济。加快创建非公经济示范市。完善扶持民营经济的政策，支持民间投资进入基本公共服务领域，参与交通、环保、燃气、供电、供水和文教卫体等经营性社会事业项目建设。加大对民营高新技术企业的支持力度。支持民营企业二次创业，扩大投资，加快发展。大力推进全民创业，壮大民营经济规模。

推进财政金融创新。继续推进“引银入玉”，支持桂林银行玉林分行、柳州银行玉林分行加快筹建工作，积极引进上海浦发银行等全国性银行。深化农村金融改革，推动农村信用合作社改制。加快发展小额贷款公司、融资性担保公司。支持玉柴组建财务公司。实施上市后备企业培育工程，推动企业上市融资。开展企业债券融资、信托融资。

加强区域合作交流。加快创建承接产业转移示范市。推进会展中心二期工程建设，办好玉博会、药博会、陶博会等展会，不断扩大开放合作平台效应。全面融入北部湾经济区开放开发，加快龙潭产业园建设，大力发展临港产业。积极参与西江经济带建设，合作共建桂东承接产业转移示范区，打造承接产业转移示范走廊。发挥海峡两岸（广西玉林）农业合作试验区的平台作用，深化玉台合作。加大面向珠三角特别是香港的招商引资力度。

发展提升开放型经济。实行更加积极主动的开放战略，加强机械、陶瓷、皮革、编织工艺品等重点行业的跟踪服务，努力扩大外贸进出口。保持一般贸易稳定增长，推动加工贸易转型升级，实现服装、鞋类、电子等产品出口新突破。加大对中小外贸企业的扶持力度，打造一批开放型龙头企业。

（九）坚持绿色发展，着力推进美丽玉林建设。

大力推进节能减排。严格水资源管理，推进节水型社会建设。严守耕地保护红线，严格土地用途管制，实现土地节约集约利用。加强能源消费管理和污染物排放总量控制，加快淘汰立窑水泥、小造纸、小塑料、小化工等落后产能。加强公共机构节能工作。

加快发展循环经济。推进有色金属、冶炼、化工、建材、服装水洗等行业的清洁生产，构建循环经济产业链。大力推进玉柴机器再制造工程，加快再生资源园区建设，打造循环经济示范企业和示范园区。支持节能低碳产业、可再生能源产业发展。

全面抓好环境整治。开展整治违法排污企业保障群众健康环保专项行动。加强重点饮用水源地的污染综合防治，推进苏烟水库饮用水源地安全保障达标建设。加快城乡污水垃圾处理设施和医疗废弃物处理设施建设，做好农村垃圾“村收镇运县处理”试点工作。开展农村面源污染综合治理、畜禽养殖污染整治和农村环境集中连片整治。加强重金属污染防治。严格化学品、核与辐射安全管理。开展PM2.5监测工作。

积极推进生态建设。加快创建生态文明示范市。继续实施“绿满八桂”造林绿化工程，重点抓好通道绿化、村屯绿化、山上造林等绿化建设，大力创建国家森林城市。推进水源湿地、自然保护区、森林公园和生态恢复工程建设。加快城镇园林绿化建设，积极创建国家园林城市。开展生态县（市、区）、绿色学校、绿色社区、绿色医院创建活动。

（十）坚持统筹兼顾，着力推进和谐社会建设。

优先发展教育。继续推进城区中小学校建设大会战，开工建设玉林师院附中新校区、玉州区金港小学、北流市第三中学新校区、兴业县实验小学等一批学校。加快推进学前教育三年行动计划。大力开展农村义务教育薄弱学校改造，促进城乡教育均衡发展。积极发展特殊教育，健全学生资助体系，促进教育公平。推进普通高中特色化、多样化发展。深入实施新一轮职业教育攻坚五年计划。支持玉林师范学院加快发展。

加快发展文化事业。加快创建特色岭南文化示范市。大力弘扬广西精神和玉林精神。加快市文化艺术中心、市博物馆等公共文化设施建设，力争开工建设王力博物馆、市图书馆新馆、市档案馆新馆。继续实施广播电视“村村通”工程和农村公益电影放映工程。扶持玉林市演艺公司等改制院团做强做大。实施文艺精品创作工程，开展文艺创作奖评选工作，推出一批文学、舞台剧、音乐、曲艺、杂技、美术等优秀文艺作品。

强化科技人才工作。引导支持骨干企业建立研发机构，促进科技合作交流和科技成果转化。加强科学知识普及，开展全民发明创造活动。推进科技特派员工作。深入实施市长质量奖。加快人才发展体制机制改革，落实人才政策，大力引进急需的科技人才和紧缺人才。加快推进人才小高地建设，打造高层次人才创新基地。统筹抓好各类人才队伍建设。

不断提高人民健康水平。深化医药卫生体制改革。推进全民医疗保障制度建设，城镇职工、城镇居民和新农合等三项基本医疗保险参保率稳定在96%以上。开展重大疾病保障工作。实施重大公共卫生项目，加强手足口病、流感等疾病防控工作。继续实施防治艾滋病攻坚工程。开展公立医院改革，组建市医疗集团。坚持计划生育基本国策，促进人口长期均衡发展。广泛开展全民健身活动，提高竞技体育综合实力和人民身体素质。

努力保持物价基本平稳。加强生产供给，增加粮食、蔬菜、食用油、猪肉等重要商品储备。健全价格调节基金制度。加强民生价格和收费监管。完善政府价格调整补贴机制。

积极推进社会管理创新。加快创建社会管理创新示范市。推进“六五”普法，提高全民法制意识。扩大法律援助覆盖面。加快城乡社区自治和服务组织建设，健全基层管理服务体系。创新流动人口和特殊人群服务管理。健全重大决策社会稳定风险评估机制，完善突发性公共事件应急管理体系。发挥“一大五小”社会服务管理平台作用，做好矛盾纠纷源头治理和信访案件化解工作，促进社会和谐稳定。

加强社会治安综合治理。大力推进“平安玉林”、“法治玉林”建设，推动“天网工程”向农村延伸，完善立体化社会治安防控体系。实施城乡社区警务战略，推进社区（农村）平安共建。加大对违法犯罪行为的打击力度。积极探索具有地域特点的农村社会治安防控工作新机制。

切实抓好安全生产。深化“打非治违”专项行动，加强重点地区、重点行业的安全整治。强化公共安全体系和企业安全生产基础建设，遏制重特大安全事故。实施食品安全行动计划，加强食品药品安全监管，提高食品药品安全保障水平。

做好国防建设工作。积极支持驻玉部队建设，进一步加强国防教育、国防动员、国防后备力量建设和人民防空工作。大力推进双拥共建活动。

充分发挥工会、共青团、妇联等群团组织的重要作用。统筹做好民族、宗教、外事、侨务、对台事务、统计、人防、气象、防震、水文、测绘、无线电管理、方志、档案、老龄等工作。

我们将一如既往地把解民忧、惠民生作为各项工作的出发点与落脚点，筹措资金50.47亿元，继续推进十项惠民工程。实施社保惠民工程。投资10.42亿元，推进城乡居民社会养老保险、城乡居民最低生活保障、五保村建设等项目，启动社会养老服务体系建设试点和社会养老服务项目建设。实施健康惠民工程。投资18.5亿元，推进新型农村合作医疗、地中海贫血防治、免费孕前优生健康检查等项目。实施教育惠民工程。投资2.5亿元，推进中等职业教育培训、扩大农村学前教育资源、学生资助等项目。实施强基惠民工程。投资3.66亿元，推进贫困村道路、小型农田水利重点县建设、农村安全饮水解困工程等项目。实施安居惠民工程。投资6亿元，推进城镇公共租赁住房和廉租房建设、城市棚户区和国有工矿棚户区改造、农村危房改造等项目。实施土地整治惠民工程。投资1.5亿元，推进农业综合开发土地治理、基本农田整治等项目。实施农补惠民工程。投资4.56亿元，推进农资综合补贴、农机具购置补贴、粮食作物良种补贴等项目。实施生态惠民工程。投资2.25亿元，推进森林生态效益补偿基金、“绿满八桂”造林绿化、农村沼气池建设、农村环境集中连片整治等项目。实施文化惠民工程。投资4000万元，推进村级公共服务中心示范、公共文化场所免费开放、农民体育健身等项目。实施通畅惠民工程。投资6750万元，推进36个建制村硬化路建设。
